# Liver, spleen, pancreas and kidney involvement by human fascioliasis: imaging findings

**DOI:** 10.1186/1471-230X-4-15

**Published:** 2004-08-04

**Authors:** Mohammad Reza Zali, Tahereh Ghaziani, Saeed Shahraz, Azita Hekmatdoost, Ali Radmehr

**Affiliations:** 1 Research Center for Gastroenterology and Liver Diseases, Shaheed Beheshti University of Medical Sciences, PO Box 19857, 7th floor, Taleghani Hospital, Tabnak Ave, Tehran, Iran; 2 Department of Radiology, Shariati Hospital, Tehran University of Medical Sciences, North Kargar Ave, Tehran-Iran; 3 Address for correspondence Unit No 14, third floor, NO 8, Hakami closed, Motahari street, Tehran, Iran

**Keywords:** Fasciola hepatica, eosinophilia, imaging

## Abstract

**Background:**

Fasciola hepatica primarily involves the liver, however in some exceptional situations other organs have been reported to be involved. The ectopic involvement is either a result of Parasite migration or perhaps eosinophilic reaction.

**Case presentation:**

Here we report a known case of multiple myeloma who was under treatment with prednisolone and melphalan. He was infected by Fasciola hepatica, which involved many organs and the lesions were mistaken with metastatic ones.

**Discussion:**

Presented here is a very unusual case of the disease, likely the first case involving the pancreas, spleen, and kidney, as well as the liver.

## Background

Human fascioliasis, a commonplace infection caused by a leaf-shaped Trematode Fasciolahepatica affects a human host by chance [1-4]. It seems that the rate of infection is increasing in many countries worldwide [1,5]. Human fascioliasis has to be differentially diagnosed from such hepatic and biliary diseases as acute hepatitis, neoplasm, visceral toxocariasis, biliary tract diseases, hepatic amebiasis, and infection with other liver flukes like schistosomiasis [2,5]. Diagnosis of the disease is achieved by locating the ova either in feces or duodendal drainage or by [7-9]. Imaging techniques proved to be the most useful method for confirming the diagnosis and also the follow-up of fascioliasis [6,8,10]. Small, often peripheral, nonenhancing, hypo dense nodules with tortuous, linear, branching tracts in CT scans, which decrease in size after successful therapy, are highly suggestive of the disease [8-12]. Immature flukes can produce ectopic masses or abscesses in various locations and during the acute phase of the disease, other structures such as subcutaneous tissue, heart, lungs, pleura, abdominal wall, brain, cecum, epididymis, and stomach can be involved [2,8,9]. In a particularly unusual report, direct peritoneal involvement with granuloma formation has been reported [1]. Eosinophilic reactions can be exhibited in the body as pleuritis and pericarditis [7,9]. Here, we present an extremely unusual radiologic incident of fascioliasis involving multiple organs.

## Case presentation

In Dec 2001, a 52 year old man, who was a known case of Multiple Myeloma (MM), was presented to one of our affiliated hospitals with persistent right upper quadrant and epigastric pain, and anorexia for a period of 1 month. At the time of admission, the patient had been receiving prednisolone and melphalan for his MM, which was currently in remission. His recent condition began with tongue and facial edema two weeks before appearance of the abdominal pain.

Upon physical examination, mild epigastric tenderness and a palpable liver were found. Neither icterus nor any positive sign of cardiopulmonary abnormalities were noted. Additionally, the patient did not have a fever and his peripheral lymph nodes were not enlarged. Initial laboratory findings were as follows: a hemoglobin of 10.6 g/dL, white blood cell count of 10,800/mm3 with 18% eosinophils, and a sedimentation rate of 90 mm at the end of the first hour. The total bilirubin was 0.5 mg/dL (0.2–0.8 mg/dl), alanine aminotranferase (ALT) 48 IU/L (0–40 IU/L), aspartate aminotransferase (AST) 46 IU/L (0–40 IU/L), and alkaline phosphatase (Alk P) 651 IU/L (60–140 IU/L). The eosinophilia fluctuated between 12 to 55 percent in various tests performed during the time period in question, with no unique patterns noted. Serum electrophoresis showed a monoclonal spike in the gamma region. Specific enzyme-linked immunosorbant assay (ELISA) produced a positive result for Fasciola hepatica, while the test was negative for Toxocara canis. Serologic tests for the presence of hepatitis A, B, and C viruses were negative. Blood and urine cultures were found to be sterile. Other laboratory studies, including repeated stool examinations for ova and parasites, showed no abnormalities. Chest x-rays did not demonstrate any parenchymal or pleural abnormality. Abdominal ultrasonography showed a mild hepatomegaly with multiple hypoechoic lesions in the liver. A CT scan revealed multiple but poorly defined, hypodense lesions in the liver, and a completely enlarged pancreas with mild bilateral pleural reaction, suggesting metastatic cancer (Fig. 1). In the search for a potential malignancy, diagnostic laprascopy was performed, which revealed the presence of white-colored lesions ranging from 1 to 3 cm in diameter on the surface of both lobes of the liver with mild ascites. Multiple liver and peritoneal biopsy specimens revealed fibrinoid necrosis, associated with granulomatous reaction and a high concentration of eosinophils in the liver, (Fig. 2) accompanied by markedly inflamed peritoneal tissue with eosniphilic infiltration. No malignant cells were identified and no evidence of extramedulary plasmocytoma was found. Specific staining for fungal organisms and acid-fast bacilli were negative. The ascitic fluid also had a high level of eosinophils. Endoscopic retrograde cholangiopancreatography (ERCP) failed to show any filling defect within the biliary tree. Furthermore, the patient underwent bone marrow aspiration that only indicated high eosniophilic infiltration. The patient was placed on albendazole (400 mg twice daily for 1 week). The treatment was well tolerated and the abdominal pain was improved rapidly. At the time of discharge, the patient was in good clinical condition. During a follow-up visit two months later, a second CT unexpectedly showed not only an increase in the number and size of the hypodense lesions in the liver, but also the extension of lesions into the pancreas, the spleen and both kidneys (Fig. 3). No evidence of peripheral enhancement of the hepatic lesions or ascites was documented. The patient was still experiencing upper quadrant pain on the right side. Laboratory investigations produced a white blood cell count of 7200/mm3 with 16% eosinophilia. Repeated stool examinations failed to identify ova and parasites. The patient was given triclabendazole (10 mg/kg, bid for two days). As recommended, the patient had another follow-up CT scan three months later. At that time all of his symptoms were resolved. Follow-up CT scans revealed a considerable improvement in the number and size of the lesions. At the time of the CT scan, all of his symptoms were resolved (Fig. 4A, B). At this time the WBC was 6000/mm3 with 6% eosinophils. After 5 months and in the last CT, the lesions had almost disappeared completely (Fig. 4C, D).

## Discussion

While fascioliasis is a well-known human parasite, it sometimes produces unusual characteristics that may influence a clinician to misdiagnose the condition.

In the vast majority of cases, the diagnosis is difficult in both acute and chronic phases and some important conditions such as liver abscesses and metastasis cannot be easily differentiated from fascioliasis [1,13]. Interestingly, the larvae is able to migrate to a number of ectopic locations such as subcutaneous areas, intestines, pleura, lungs, abdominal wall, brain, cecum, epididymis, stomach, pericardial, or cerebral sites, producing very unique clinical manifestations [8,9]. Serologic testing, when performed by an enzyme linked immunoabsorbent assay (ELISA) for the detection of antibodies specific to the parasite is nearly one hundred percent sensitive and specific. Thus ELISA may be used to confirm the diagnosis in acute and chronic phases [7-9].

Among imaging tools, ultrasonography is of little diagnostic value during the acute phase, while a contrast-enhanced CT scan can be very useful for diagnosis [9,17]. In CT images one can find two distinct kinds of lesions: single or multiple hypodense nodular areas caused by deposition of the parasite (abscess-like lesions) and tunnel-like branching or tortuous hypodensity which is created as the result of parasite migration through the liver and is highly suggestive of the disease [9,17]. If peripheral tortuous lesions are present, hepatic fascioliasis should be the primary diagnostic consideration [14].

As clinical and laboratory findings of fascioliasis may easily be confused with many other conditions, a high index of suspicion is required to establish a correct diagnosis [9,17]. Both CT scan and ultrasonography can be helpful in evaluating the response to treatment [15].

The ingested metacercariae of Fasciola hepatica penetrate the intestinal wall and migrate through the peritoneal cavity to reach the liver. However, ectopic migration to other locations is one of the strangest manifestations of the infection [8,9]. The precise route of migration toward ectopic sites is unclear but most often occurs in the acute stage of the disease [9]. In addition, a syndrome of eosinophilic reaction without direct parasitic involvement may accompany acute fascioliasis [9]. Ascites is not a common finding in fascioliasis, but it is not unheard of, as the peritoneum is the usual route of migration of the parasite towards the liver. Kabaalioglu A. et al reported mild splenomegaly and enlargement of the left rectus abdominalis muscle in hepatic fascioliasis [17]. In a study the authors found that among pleuro-pulmonary diseases, parenchymal infiltrates resembling the Loeffler syndrome and pleural effusion were the most common radiological features [8]. Radiologic manifestations of fascioliasis in a case with peritoneal involvement have been described as low-density lesions in the mesentery [18]. While the radiologic presentations of the disease are contradictory, reporting any new set of images related to the unusual organ involvement is of paramount importance. The case presented here is probably the first report of fascioliasis with spleen, pancreas, and kidney involvement. Migration of Fasciola hepatica to the spleen and pancreas and perhaps the kidneys was considered as the cause of the imaging findings in this patient, however hypersensitivity reaction to Fasciola antigens might also be implicated. Pathology examination to confirm the ectopic locations of the involvement was not performed, however improvement of the lesions on follow-up imaging studies after treatment defended the involvement of those regions by the parasite The compromised immunity of the patient linked to the use of immunosuppressive medicines could partly explain the extensive extra-hepatic involvement of the disease. The patient responded fully to Triclabendazole and is now healthy.

## Pre-publication history

The pre-publication history for this paper can be accessed here:


